# Poles Apart: The “Bipolar” Pteropod Species *Limacina helicina* Is Genetically Distinct Between the Arctic and Antarctic Oceans

**DOI:** 10.1371/journal.pone.0009835

**Published:** 2010-03-23

**Authors:** Brian Hunt, Jan Strugnell, Nina Bednarsek, Katrin Linse, R. John Nelson, Evgeny Pakhomov, Brad Seibel, Dirk Steinke, Laura Würzberg

**Affiliations:** 1 Department of Earth and Ocean Sciences, University of British Columbia, Vancouver, British Columbia, Canada; 2 Department of Genetics, La Trobe Institute for Molecular Science, La Trobe University, Bundoora, Australia; 3 British Antarctic Survey, Cambridge, United Kingdom; 4 Fisheries and Oceans Canada, Sidney, British Columbia, Canada; 5 Department of Biological Sciences, University of Rhode Island, Kingston, Rhode Island, United States of America; 6 Biodiversity Institute of Ontario, University of Guelph, Guelph, Ontario, Canada; 7 Zoological Institute and Museum, University of Hamburg, Hamburg, Germany; Mt. Alison University, Canada

## Abstract

The shelled pteropod (sea butterfly) *Limacina helicina* is currently recognised as a species complex comprising two sub-species and at least five “forma”. However, at the species level it is considered to be bipolar, occurring in both the Arctic and Antarctic oceans. Due to its aragonite shell and polar distribution *L. helicina* is particularly vulnerable to ocean acidification. As a key indicator of the acidification process, and a major component of polar ecosystems, *L. helicina* has become a focus for acidification research. New observations that taxonomic groups may respond quite differently to acidification prompted us to reassess the taxonomic status of this important species. We found a 33.56% (±0.09) difference in cytochrome *c* oxidase subunit I (COI) gene sequences between *L. helicina* collected from the Arctic and Antarctic oceans. This degree of separation is sufficient for ordinal level taxonomic separation in other organisms and provides strong evidence for the Arctic and Antarctic populations of *L. helicina* differing at least at the species level. Recent research has highlighted substantial physiological differences between the poles for another supposedly bipolar pteropod species, *Clione limacina*. Given the large genetic divergence between Arctic and Antarctic *L. helicina* populations shown here, similarly large physiological differences may exist between the poles for the *L. helicina* species group. Therefore, in addition to indicating that *L. helicina* is in fact not bipolar, our study demonstrates the need for acidification research to take into account the possibility that the *L. helicina* species group may not respond in the same way to ocean acidification in Arctic and Antarctic ecosystems.

## Introduction

Over the past 200 years the world's oceans have absorbed approximately one third of the total carbon dioxide (CO_2_) released into the atmosphere by human activities [1]. This CO_2_ uptake is causing profound changes to seawater chemistry, including a reduction in pH (i.e., ocean acidification) and a reduction in the saturation state of calcium carbonate (CaCO_3_) [2]. The latter is critical to the formation of CaCO_3_ skeletal structures by a wide range of marine organisms, including molluscs, corals, echinoderms and crustaceans, as their calcification rates are directly related to the CaCO_3_ saturation state of seawater [3]. Decreasing CaCO_3_ saturation levels are of particular concern for organisms that build their skeletons out of aragonite, a metastable form of CaCO_3_ that is ∼50% more soluble than calcite, and for organisms in the polar regions where CaCO_3_ undersaturation, and hence skeletal dissolution, is expected to occur first [4]. Recent projections are that localised aragonite undersaturation of Arctic surface waters may occur within a decade [5], while the surface waters of the Southern Ocean (Antarctic) may begin to become aragonite undersaturated by as early as 2030 [6].

**Figure 1:**
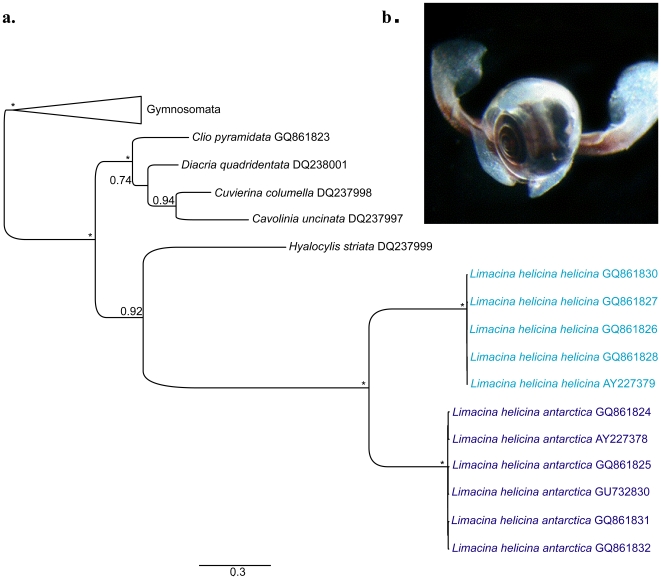
Genetic distance between Arctic and Antarctic *Limacina helicina*. **a.** *L. helicina antarctica* from the Lazarev Sea, Antarctic (photo: R. Giesecke); **b.** Bayesian tree depicting the phylogenetic relationships of pteropod molluscs. The genetic distance between cytochrome *c* oxidase subunit I (COI) gene sequences of *L. helicina* individuals was 0.15±0.06% and 0.60±0.07% within the Arctic (*L. helicina helicina* forma *helicina*) and Antarctic (*L. helicina antarctica*) respectively, but 33.56±0.09% between poles. Support is indicated as posterior probabilities above nodes (* indicate 1.0 support) and bootstraps from a maximum likelihood analysis below (* indicate 100% support). The scale bar represents substitutions per site. GQ861824 and GQ861825 from the Amundsen Sea; GQ861831, GQ861832 and GU732830 from the vicinity of South Georgia; GQ861826/27/28/30 from the Beaufort Sea; AY22739 and AY227378 from [12].

Aragonite-shelled (thecosome) pteropods, pelagic swimming sea snails sometimes referred to as sea butterflies, occur in all oceans but are particularly abundant in the polar regions [7], [8]. Here they are principally represented by what is considered to be a bipolar species, *Limacina helicina* (Phipps 1774) (Figure 1a). Because of its aragonite shell and polar distribution, *L. helicina* may be one of the first organisms affected by ocean acidification, and it is therefore a key indicator species of this process [3]. *L. helicina* is a major component of the polar zooplankton. It can comprise >50% of total zooplankton abundance (number of individuals per unit volume) and it plays a significant ecological role as a phytoplankton grazer and prey species for zooplankton and fish, while also contributing substantially to carbonate and organic carbon flux [8]. As one of the organisms most vulnerable to ocean acidification, and a key component of polar ecosystems, *L. helicina* has become a focal point for research on acidification impacts [3], [9].

Currently, northern and southern hemisphere *L. helicina* are listed as the sub-species *L. helicina helicina* and *L. helicina antarctica* respectively. In addition, the taxonomic category “forma” has been applied to designate at least three morphotypes of *L. h. helicina* (*acuta*, *helicina* and *pacifica*) and two morphotypes of *L. h. antarctica* (*antarctica* and *rangi*). These forms typically have different geographical ranges but it remains unclear as to whether “forma” represent morphological responses to different environmental conditions or are indeed taxonomically distinct, and if the latter, their level of taxonomic separation [10]. Recent findings show that the response of organisms' calcification rates to acidification can vary markedly between taxonomic groups [11]. It is hypothesised that this varied response is due to physiological differences, occurring even at the species level. In the absence of a detailed understanding of, and ability to measure, the physiological processes controlling calcification rates, correct taxonomic data are critical for quantifying acidification impacts.

Given the importance of *L. helicina* as an indicator of ocean acidification there is an urgent need for research that will resolve the taxonomic status of the *L. helicina* group. Molecular techniques represent an appealing route to take as they avoid the potential confusion resulting from environmentally induced morphological plasticity. The cytochrome *c* oxidase subunit I (COI) gene has been demonstrated to be well suited to gastropod phylogenetics [12]. Based on a single specimen of each, Remigio and Hebert [12] provided initial evidence for the genetic separation of *L. h. helicina* and *L. h. antarctica*. Here, we build upon their study and use COI sequences from multiple specimens of the Arctic *L. h. helicina* forma *helicina* and the Antarctic *L. h. antarctica* to quantify genetic distance within and between these regions with the specific aim to assess the bipolar status of the *L. helicina* species group.

## Results and Discussion

We found a 33.56% (±0.09) difference in COI sequences between the Arctic *L. h. helicina* forma *helicina* and the Antarctic *L. h. antarctica* (Figure 1b). This degree of separation is sufficient for ordinal level taxonomic separation in other organisms [13] and convincingly demonstrates that *L. helicina* is not bipolar, but that the Arctic and Antarctic populations differ at least at the species level. Our results support Remigio and Hebert [12] in identifying *L. helicina* as a rate-accelerated lineage within pteropods (Figure 1b). A conservative divergence time estimate of 31 Ma (95% HPD interval 12–53 Ma) for Arctic and Antarctic *L. helicina*, indicates that they have undergone rapid independent evolution since the establishment of cold water provinces in the early Oligocene.

Our results show the need for a revision of the taxonomic status of the *L. helicina* species group. The high degree of separation at what is considered the sub-species level, suggests that COI sequences analysis may also provide an effective means to clarify the relationships between the “forma” of both *L. h. helicina* and *L. h. antarctica*. Our study only included one form of *L. h. helicina* (forma *helicina*). In the case of the Antarctic sub-species, forma was not determined for any of the specimens analysed. Based on known biogeographic distributions the Amundsen Sea specimens were most likely forma *antarctica* [10], while analysis of the South Georgia net samples indicated that only forma *antarctica* were present. Although it is possible that the South Georgia specimens sequenced were forma *rangi*, the high COI sequence similarity between Antarctic samples demonstrated that specimens were closely aligned. It remains to be determined whether this similarity was form specific, or whether forma are indeed morphotypes and not genetically distinct.

As highlighted in the introduction, due to unique physiologies, the response of organisms to ocean acidification may vary even at the species level. A recent study comparing the locomotor abilities of another supposedly bipolar pteropod species, *Clione limacina*, identified significant differences in the aerobic capacity of Arctic and Antarctic forms, associated with neuromuscular and mitochondrial composition [14]. Given the substantial genetic divergence between Arctic and Antarctic *L. helicina* populations observed in our study, similarly large physiological differences may exist between the poles for the *L. helicina* species group. Therefore, in addition to the taxonomic implications, our study demonstrates the need for acidification research to take into account the possibility that the *L. helicina* species group may not respond in the same way to ocean acidification in the Arctic and Antarctic. Physiological differences between taxa coupled with differences in the processes and rates of acidification at the poles [4], [5], [6], brings to light the possibility that differences in acidification impacts in the Arctic and Antarctic may extend beyond species to the ecosystem level.

## Materials and Methods

Specimens of the Antarctic *Limacina helicina antarctica* were obtained from the Amundsen Sea and the vicinity of South Georgia Island (Figure 1b). The forma of these specimens was not determined. Specimens of the Arctic *Limacina helicina helicina* were identified as forma *helicina* and were obtained from the Beaufort Sea. Full locations and station data are available on Barcode of Life Data systems (BOLD)/GenBank. Extraction, amplification and sequencing followed standard DNA barcoding protocols [15], [16]. DNA was also extracted using the high salt method [17]. PCR amplifications were performed using the standard Folmer [18] primers and sequencing was carried out by Macrogen (Korea). New sequences have been deposited in BOLD/GenBank (Accession numbers GQ861824–861832, GU7328230). The length of *L. helicina* sequences varied from 528 bp to 618 bp. This variation reflects difficulty in amplifying the fragments. Alternative COI primers, a combination of standard primers [18] and mini-barcode primers yielding two overlapping fragments [19], had to be used in addition to recover these shorter sequences. The published sequences of Remigio and Hebert [12] for single specimens of *L. h. helicina* and *L. h. antarctica* were included in subsequent calculations of genetic distance.

The K2P model [20] of sequence evolution was used to calculate the genetic distance for *L. h. helicina* and *L. h. antarctica* both within and between regions, (i.e., Arctic and Antarctic) using PAUP 4.0b10 [21]. The genetic distance between COI sequences of five individuals collected from the Arctic was 0.15±0.06%, whilst the genetic distance between COI sequences of six individuals collected from the Antarctic was 0.60±0.07%. Genetic distance between individuals collected from the two regions was 33.56±0.09%.

Bayesian analyses were conducted using BEAST v1.4.8 [22], using a SRD06 nucleotide model [23]. Analyses were run with both strict clock and uncorrelated log-normal relaxed clock [24] models, with the mean substitution rate fixed to 1.0. A Yule prior on branching rates was employed [24]. Gymnosomata and Thecosomata were assumed to be reciprocally monophyletic [25]. Two independent MCMC analyses were run for each parameter set. Acceptable mixing and an appropriate ‘burnin’ was determined using Tracer v1.4.1 [26]. Each analysis was conducted for 20 million generations sampling every 1000 generations. The Bayes factor [27] was used to compare strict and relaxed clock models as implemented in Tracer v1.4.1. The uncorrelated log-normal relaxed clock model was preferred with a Bayes Factor (natural log) of 20.9±0.2.

Phylogenetic maximum likelihood analyses were performed with RAxML v.7.0.4 [28]. All searches were completed with the GTRMIX option and bootstraps were calculated with 1000 replicates. To obtain a minimum divergence time estimate of *L. helicina* from Arctic and Antarctic regions we also analysed the data within BEAST v1.4.8 (using a SRD06 nucleotide model and an uncorrelated log-normal relaxed clock model) using a fixed calibration date of 58.7 Ma on the divergence of *Limacina* (Limacinidae) and *Hyalocylis* (Creseinae) [29]. *L. mercinensis* is the oldest known limacinoid fossil from the Thanetian (58.7±0.2-55.8±0.2 Ma) [30]. The oldest known Creseinae fossils are from the Ypresian (55.8±0.2-48.6±0.2 Ma) [29]. Therefore the Limacinoidea and Cresinae lineages must have diverged prior to the Thanetian. The age of Thecosomata was constrained to be less than 65 Ma as the group is understood to have evolved in the Cenozoic [31].

In recent classification [32] the family Cavoliniidae contains the subfamilies Cavoliinae, Clioinae, Cuvierininae and Creseinae. In contrast, in our topology the Cavoliniidae is paraphyletic, with a sister taxon relationship between *Hyalocylis* (Creseinae) and *Limacina*. This relationship was further supported by the possession of a shared indel by both taxa not present in any of the other species sequenced.

## References

[1] Sabine CL, Feely RA, Gruber N, Key RM, Lee K, et el (2004). The Oceanic Sink for Anthropogenic CO_2_.. Science.

[2] Feely RA, Sabine CL, Lee K, Berelson W, Kleypas J (2004). Impact of Anthropogenic CO_2_ on the CaCO_3_ System in the Oceans.. Science.

[3] Fabry VJ, Seibel BA, Feely RA, Orr JC (2008). Impacts of ocean acidification on marine fauna and ecosystem processes.. ICES J Mar Sci.

[4] Orr JC, Fabry VJ, Aumont O, Bopp L, Doney SC (2004). Anthropogenic ocean acidification over the twenty-first century and its impact on calcifying organisms.. Nature.

[5] Steinacher M, Joos F, Frölicher TL, Plattner GK, Doney SC (2009). Imminent ocean acidification in the Arctic projected with the NCAR global coupled carbon cycle-climate model.. Biogeosciences.

[6] McNeil BI, Matear RJ (2008). Southern Ocean acidification: A tipping point at 450-ppm atmospheric CO_2_.. Proc Natl Acad Sci.

[7] Lalli CM, Gilmer RW (1989). Pelagic Snails: The Biology of Holoplanktonic Gastropod Mollusks..

[8] Hunt BPV, Pakhomov EA, Hosie GW, Siegel V, Ward P, Bernard K (2008). Pteropods in Southern Ocean ecosystems.. Prog Oceanogr.

[9] Comeau S, Gorsky G, Jeffree R, Teyssié JL, Gattuso JP (2009). Impact of ocean acidification on a key Arctic pelagic mollusc (*Limacina helicina*).. Biogeosciences.

[10] van der Spoel S, Dadon JR, Boltovskoy D (1999).

[11] Fabry VJ (2008). Marine Calcifiers in a High-CO_2_ Ocean.. Science.

[12] Remigio EA, Hebert PDN (2003). Testing the utility of partial COI sequences for phylogenetic estimates of gastropod relationships.. Mol Phylog Evol.

[13] Costa FO, deWaard JR, Boutillier J, Tanasingham S, Dooh R T (2007). Biological identifications through DNA barcodes: the case of Crustacea.. Can J Fisheries Aqua Sci.

[14] Rosenthal JJC, Seibel BA, Dymowska A, Bezanilla F (2009). Trade-off between aerobic capacity and locomotor capability in an Antarctic pteropod.. Proc Natl Acad Sci.

[15] Hebert PDN, Ratnasingham S, deWaard JR (2006). Barcoding animal life: cytochrome *c* oxidase subunit 1 divergences among closely related species.. Proc Roy Soc Lond B.

[16] Ivanova N, Dewaard JR, Hebert PDN (2006). An inexpensive, automation-friendly protocol for recovering high-quality DNA.. Molecular Ecology Notes.

[17] Sambrook J (1989). Molecular Cloning: A Laboratory Manual, 2nd edn..

[18] Folmer O, Black M, Hoeh W, Lutz R, Vrijenhoek R (1994). DNA primers for amplification of mitochondrial cytochrome c oxidase subunit I from diverse metazoan invertebrates.. Molec Mar Biol Biotech.

[19] Hajibabaei M, Janzen DH, Burns JM, Hallwachs W, Hebert PDN (2006). DNA barcodes distinguish species of tropical Lepidoptera.. Proc Nat Acad Sci.

[20] Kimura M (1980). A simple method for estimating evolutionary rate of base substitutions through comparative studies of nucleotide sequences.. J Molec Evol.

[21] Swofford DL (1998).

[22] Drummond AJ, Rambaut A (2007). BEAST: Bayesian evolutionary analysis by sampling trees.. BMC Evol Biol.

[23] Shapiro B, Rambaut A, Drummond AJ (2006). Choosing appropriate substitution models for the phylogenetic analysis of protein-coding sequences.. Mol Biol Evol.

[24] Drummond AJ, Ho SYW, Phillips MJ, Rambaut A (2006). Relaxed phylogenetics and dating with confidence.. PLoS Biol.

[25] Klussmann-Kolb A, Dinapoli A (2006). Systematic position of the pelagic Thecosomata and Gymnosomata within Opistobranchia (Mollusca, Gastropoda)–revival of the Pteropoda.. J Zoo Syst Evol Res.

[26] Rambaut A, Drummond AJ (2003). Tracer version 1.2 [computer program].. http://tree.bio.ed.ac.uk/software/tracer/.

[27] Kass RE, Raftery AE (1995). Bayes factors.. J Amer Statis Assoc.

[28] Stamatakis A (2006). RAxML-VI-HPC: Maximum likelihood based phylogenetic analyses with thousands of taxa and mixed models.. Bioinformatics.

[29] Cahuzac B, Janssen AW Eocene to Miocene holoplanktonic Mollusca (Gastropoda) of the Aquitaine Basin, SW France.. Scripta Geologica. In press.

[30] Benton MJ (1993). The fossil record 2..

[31] Wägele H, Klussman-Kolb A, Vonnemann V, Medina M, Lindberg D, Ponder W (2008). Heterobranchia I. The Opisthobranchia.. Phylogeny and Evolution of the Mollusca. 383–406.

[32] Bouchet P, Rocroi JP (2005). Classification and Nomenclator of Gastropod families.. Malacologia.

